# Supplementary material for: XRN2 Is Required for Cell Motility and Invasion in Glioblastomas
Source: Cells. 2022 Apr 28;11(9):1481. doi: 10.3390/cells11091481 (PMC9100175; doi:10.3390/cells11091481)

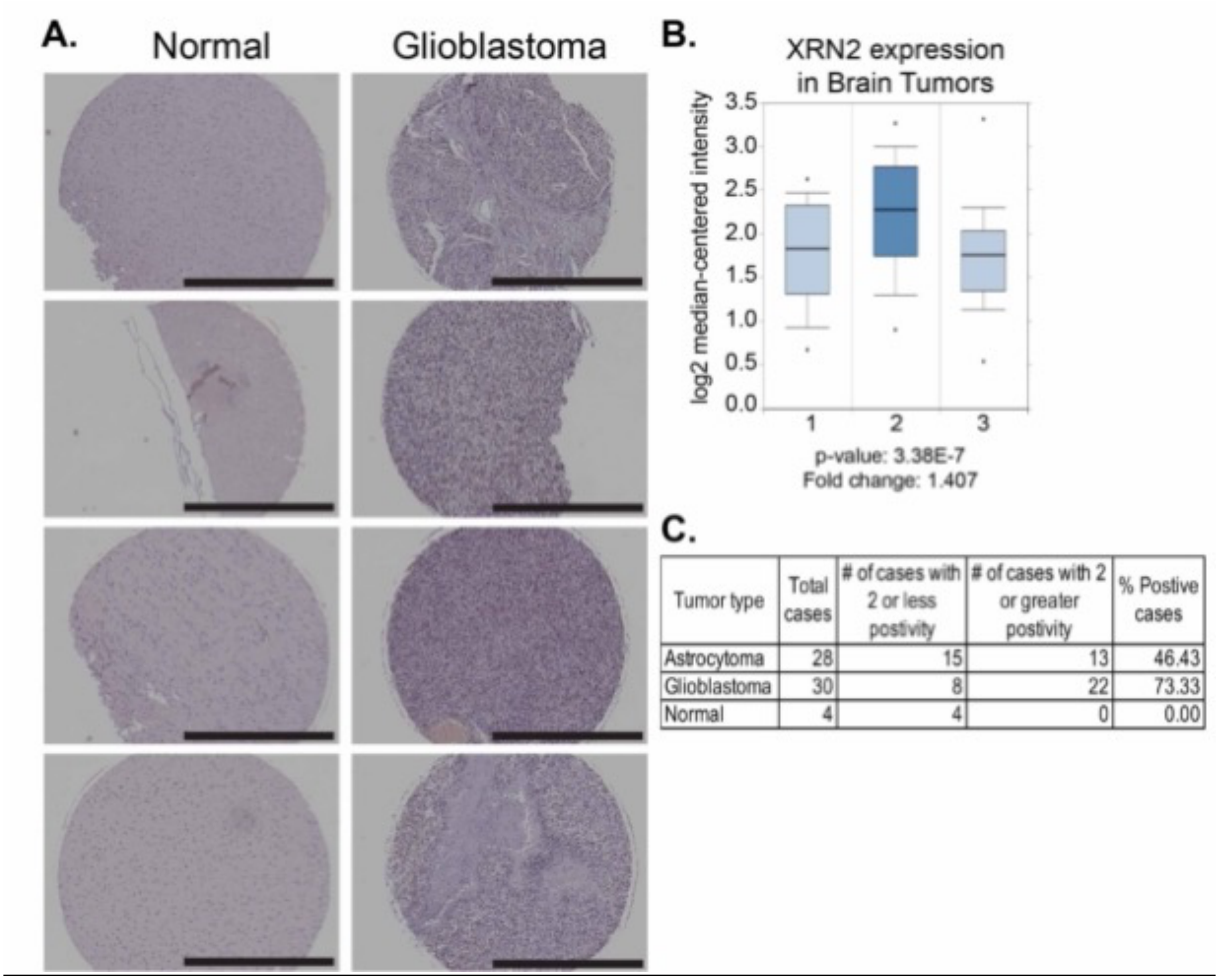

<u>Supplemental Figure S1.</u> XRN2 expression is elevated in glioblastoma. (A) H&E of tissue samples depicted in Figure 1B. Scale bar is 1000 micron. (B) XRN2 mRNA level were analyzed using the publicly accessible Oncomine database in 1) astrocytomas, 2) glioblastomas and 3) oligodendrogliomas. (C) XRN2 protein expression was measured in normal, glioblastoma and astrocytoma tissue samples.

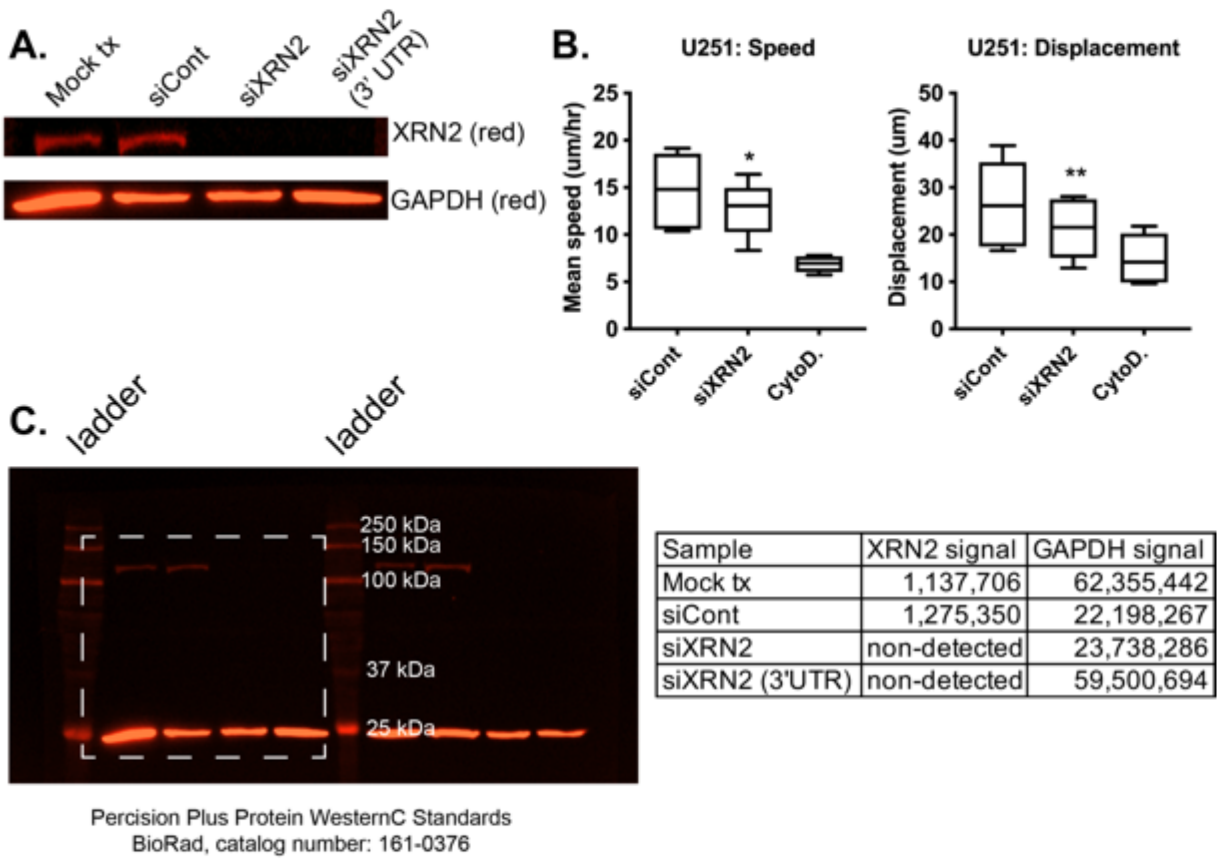

Sample	XRN2 signal	GAPDH signal
Mock tx	1,137,706	62,355,442
siCont	1,275,350	22,198,267
siXRN2	non-detected	23,738,286
siXRN2 (3'UTR)	non-detected	59,500,694

Supplemental Figure S2. XRN2 is required for cell motility
(A) Western blot of XRN2 expression in U251 cells. Cells were treated with the listed siRNAs. GAPDH is the loading control. (B) Cell motility tracking of speed and displacement of U251 H2B-GFP cells over a 6-hour imaging period with 30-miniute intervals. (C) Full blot and dosimetry readings of blot shown in A. * p-value ≤ 0.05, ** p-value ≤ 0.01. Students t test was used for statistical analysis.

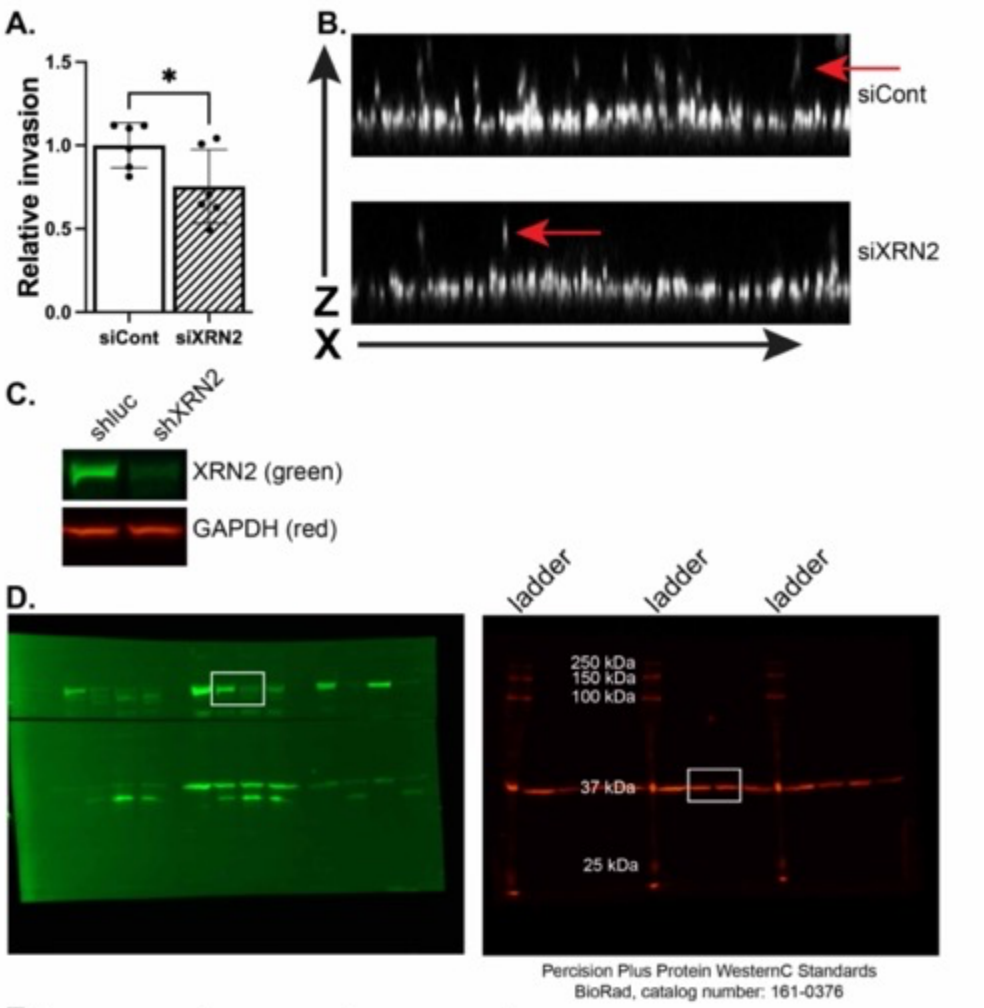

E.

	shluc	shXRN2-3640
XRN2 (green)	2,852,245	2,093,933
GAPDH (red)	15,430,812	17,971,616

Supplemental Figure S 3. Loss of XRN2 reduces G55 cell's ability to invade into matrix. (A, B) The inverted vertical invasion assay was used to monitor migration through a matrix in G55 cells that were exposed to control or XRN2 siRNAs. (C-E) Steady state levels of XRN2 was measured and quantitated by western blot analysis in G55 cell transduced with the listed shRNAs. Students t test was used for statistical analysis. *=p>0.05

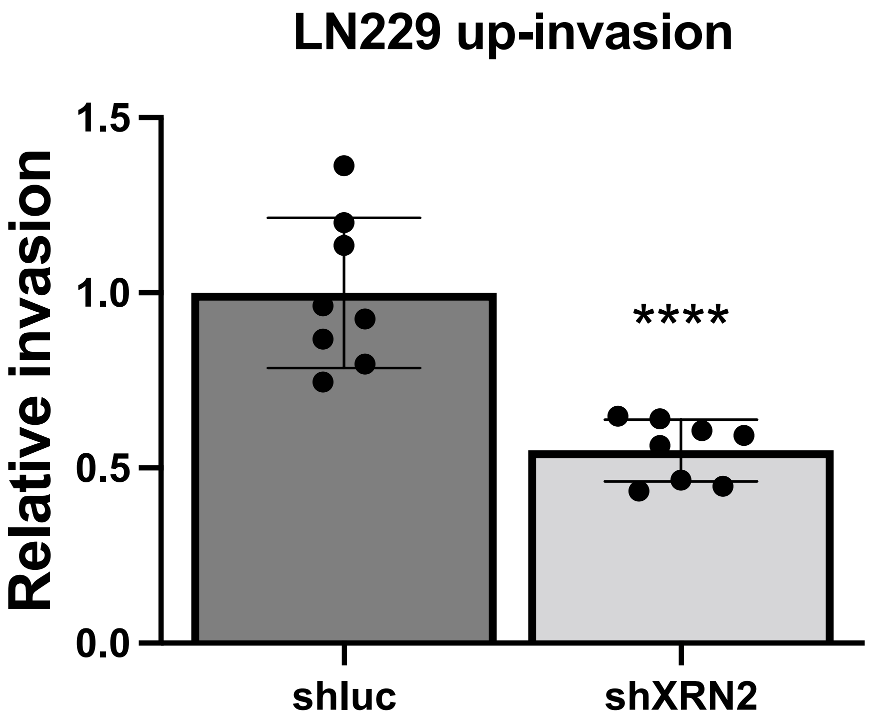

<u>Supplemental Figure S4.</u> Loss of XRN2 reduces LN229 cell's ability to invade into matrix. (A, B) The inverted vertical invasion assay (up invasion) was used to monitor migration through a matrix XRN2 positive (shluc) and XRN2 deficient (shXRN2) LN229 cells. Students t test was used to perform statistical analysis. ****=p>0.0001

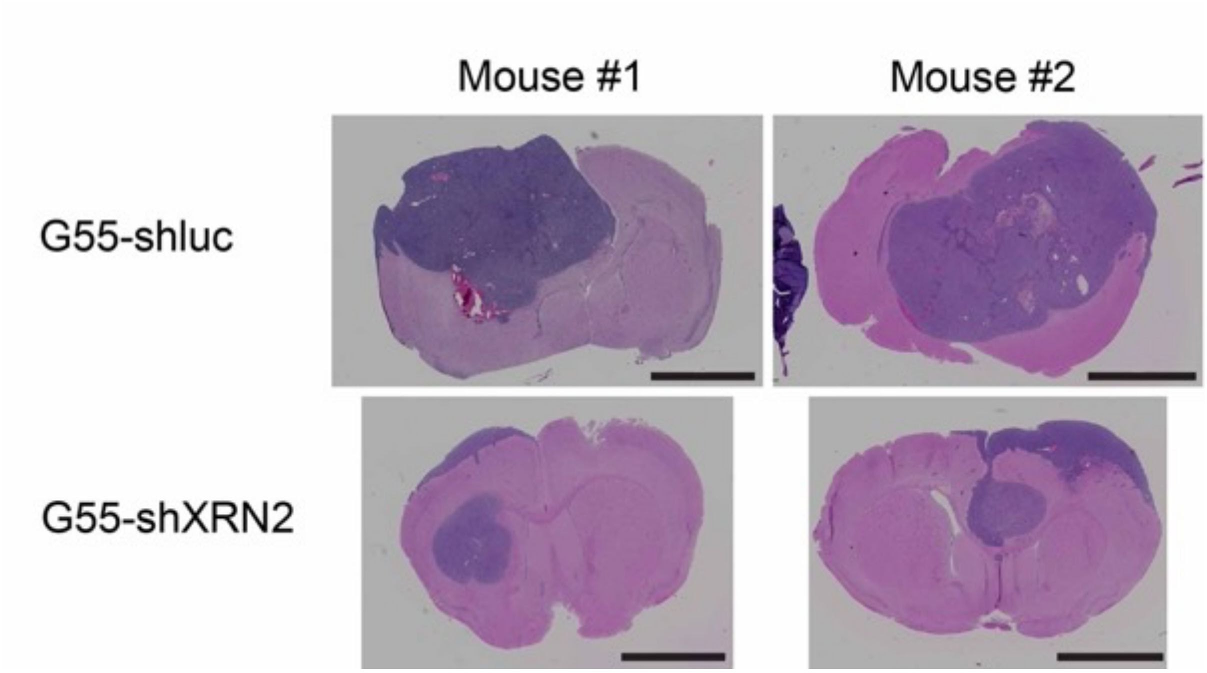

<u>Supplemental Figure S 5.</u> XRN2 deficient G55 cells form tumors with reduced volumes. Hematoxylin-eosin staining was used to detect tumor formation in mouse brains injected with control (G55-shluc) and XRN2 deficient (G55-shXRN2) G55 cells. Black scale is 3000 micron.

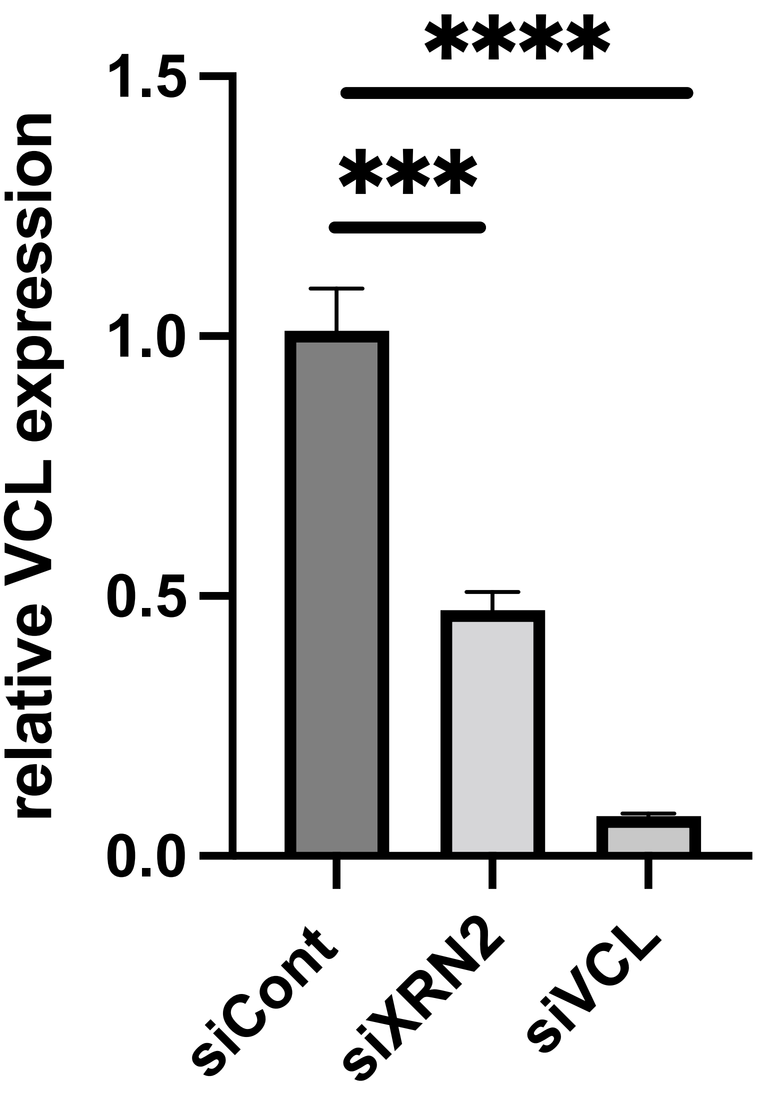

<u>Supplemental Figure S6.</u> Loss of XRN2 results in loss of VCL expression. mRNA levels of VCL were monitored in U251 cells that were exposed to control (siCont), XRN2 (siXRN2) and VCL (siVCL) siRNAs. Measurements were performed in triplicate. Students t test was used for statistical analysis. ***=p>0.001 and ****=p>0.0001

Supplemental Movie 1

<u>Supplemental Video S1.</u> Loss of XRN2 decreases cell speed and motility in U87 GBM cells. Live cell imaging was done using H2B-GFP labeled U87 cells transfected with control siRNA (left), XRN2 siRNA (middle) or exposed to cytochalasin D (right).

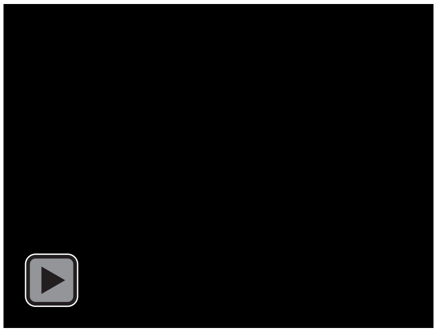

Supplement: Supplementary file 1 [file cells-11-01481-s001.zip › cells-1628157-supplementary.pdf]
